# Partnership Living Arrangements of Immigrants and Natives in Germany

**DOI:** 10.3389/fsoc.2020.538977

**Published:** 2020-11-13

**Authors:** Anne-Kristin Kuhnt, Sandra Krapf

**Affiliations:** ^1^Department of Sociology, University of Duisburg-Essen, Duisburg, Germany; ^2^Mannheim Centre for European Social Research (MZES), University of Mannheim, Mannheim, Germany

**Keywords:** cohabitation, Turkish migrants, ethnic German migrants, integration, German microcensus, single, parental home

## Abstract

This paper compares the partnership arrangements of Turkish and Ethnic German immigrants (i.e., return migrants from Ethnic German communities from predominantly Eastern European countries), the two largest migrant groups in Germany, and native Germans. Most existing analyses of migrants' partnerships focus on intermarriage, marriage formation, or union dissolution. We know only a little, however, about the prevalence of non-marital living arrangements. Given that single person households and cohabitation are widespread phenomena mainly in post-materialist societies, analyzing whether immigrants engage in these behaviors sheds light on potential adaptation processes. The analyses are based on the German Microcensus of the years 2009 and 2013, with a focus on adults in the 18–40 age group. First, we present descriptive findings on the prevalence of partnership arrangements of immigrants and native Germans. Second, we estimate cross-sectional regressions with the partnership arrangement as the outcome variable in order to control for compositional differences between immigrant groups with respect to education. Our results show that while the vast majority of first-generation immigrants are married, the share of married natives is considerably smaller. Living in an independent household without a partner and cohabitation are rare phenomena among immigrants. By contrast, about one in seven natives is cohabiting and more than one quarter is living in an independent household without a partner. The most prevalent partnership living arrangement of the Turkish second generation is living in the parental household without a partner. These results are robust after controlling for education, age, and year in the multiple regression analysis.

## Introduction and Background

International migration is an event that affects every facet of a migrant's life. While many studies in Germany and European countries have focused on the socio-economic sphere, investigating migrants' educational success (Kristen, 2014; Kuhnt, 2017), labor market behaviors (Kogan, 2011), and social well-being (Kuhnt and Wengler, 2019), an increasing number of studies acknowledge the relevance of the family domain: e.g., migrants' fertility (Milewski, 2010; Krapf and Wolf, 2015; Kreyenfeld and Krapf, 2017; Kulu et al., 2017), marriage formation (González-Ferrer, 2006; Kalter and Schroedter, 2010; Weißmann and Maddox, 2016), cohabitation (Hannemann and Kulu, 2014; Hannemann et al., 2020), and divorce behavior (Milewski and Kulu, 2014). However, information about the prevalence of living without a partner or of cohabiting among immigrants in Germany is rare (e.g., Naderi, 2008). This is surprising, as Germany is one of the countries with the highest proportions of migrants in Europe: The share of the population who did not acquire German citizenship by birth, or who have a parent who was not born a German citizen, was 25.5% (20.8 million individuals) in 2018 (Destatis, 2019b). Investigating the partnership patterns of migrants – not only for Germany—is relevant for two reasons. First, cohabitation (Noack et al., 2013) and single person households (Klinenberg, 2012; Eurostat, 2020) have been established as widespread phenomena mainly in Western societies. Analyzing whether migrants engage in these behaviors sheds light on adaptation processes—especially if the migrants come from countries with more traditional family values and behaviors. Second, family decisions, and especially the timing of these decisions, determine individual opportunities in life. For example, early marriage is often followed by early childbearing and lower levels of labor market participation among women (Pienta, 1999), and might thus increase social inequality.

This study aims to identify the partnership living arrangements of Turkish migrants, Ethnic German migrants (i.e., return migrants from predominantly Eastern European countries), and native Germans. In the first step, we compare the prevalence of partnership living arrangements (i.e., living without a partner in an independent household, living without a partner in the parental household, cohabitation, and marriage) across immigrant groups. Second, we examine whether compositional effects with regard to education exist. We chose to focus on Ethnic German migrants and Turkish migrants because they represent the two largest immigrant groups in Germany. Moreover, the partnership behaviors in Turkey and in the origin countries of Ethnic Germans differ significantly from those in Germany, and such differences enable us to identify potential adaptation processes. Unfortunately, to our knowledge, there is no longitudinal dataset that is large enough to analyse the partnership transitions of different immigrant groups in Germany^1^. Therefore, we provide a descriptive account of partnership arrangements by origin group based on the largest cross-sectional survey in Germany, the Microcensus. We focus on individuals aged 18–40 years because young adults have more dynamic partnerships (Manning, 2020), and differences in living arrangements decline with increasing age. While the first and second generations of Turkish immigrants are investigated separately, we analyse only first-generation Ethnic German immigrants. Since most Ethnic German immigrants arrived in Germany later than many Turkish immigrants, the number of second-generation Ethnic German immigrants in the relevant age group is still too small to be analyzed as a separate group.

In the following, we provide a brief overview of the immigration context of Turkish and Ethnic German immigrants, and we discuss theoretical arguments and prior research about immigrants' partnership decisions. The rest of the paper is devoted to the empirical analyses.

### Turkish and Ethnic German Immigrants in Germany

We focus our study on Turkish and Ethnic German migrants, as they represent the two largest groups of migrants in Germany, respectively making up 13.5% (2.8 million individuals) and 12.5% (2.6 million individuals) of the country's first- and second-generation immigrants (Destatis, 2019a, p. 128). The largest inflows of Turkish immigrants occurred in the 1960s and 1970s, and were triggered by the recruitment agreements signed between West Germany and Turkey in 1961 (Oltmer, 2018). The recruitment agreements in Germany came to a halt in 1973. Since then, family reunion has been the largest driver of Turkish immigration to Germany (Bundesamt für Migration und Flüchtlinge, 2016b, 2019a). With respect to partnership living arrangements in Turkey, marriage is the most common arrangement among people aged 18–29 (68.4%), followed by living without a partner in the household (30.7%; Inglehart et al., 2014). Cohabitation still seems to be unacceptable: only 0.8% of the respondents were living with a partner outside of marriage (period 2010–2014; Inglehart et al., 2014).

Ethnic German immigrants came to Germany from a number of countries, mainly Poland, Romania, and the Soviet Union (and its successor states Kazakhstan and Russia). In these countries, Ethnic German communities had existed for many decades. After the collapse of the Soviet Union in the 1990s, a massive wave of migration to Germany took place. Being recognized as an Ethnic German immigrant guarantees full German citizenship (Hensen, 2009; Worbs et al., 2013). The migration flows of Ethnic Germans have recently slowed and will eventually come to a complete halt, as by law Ethnic German migration is impossible for individuals born in 1992 or later (Bundesamt für Migration und Flüchtlinge, 2019b). Therefore, the timeframe for investigating the partnership behavior of first-generation Ethnic German immigrants is limited. Regarding the partnership arrangements in Russia and Poland, two important countries of origin of Ethnic Germans, marriage is the most common living arrangement among individuals aged 18–29, at 48.3% in Russia and 57.5% in Poland. Living without a partner is also common, at 44.5% in Russia and 37.4% in Poland. The least common partnership arrangement is cohabitation, at 6.5% in Russia and 4.5% in Poland (Inglehart et al., 2014).

### Theoretical Considerations and Prior Research

Although we are unable to test specific hypotheses with our data, we embed our research report in several theoretical arguments. Partnership behaviors are largely the product of cultural and structural determinants (Glick, 2010). In terms of cultural determinants, two contradictory forces are at work that affect the attitudes and behaviors of immigrants from traditional countries: (primary) socialization and adaptation. The *socialization hypothesis* explains why differences in partnership behaviors between immigrants and natives in the country of destination might persist. Individuals socialized in countries with more traditional family values than Germany—such as Turkey (Voicu, 2017) and to a lesser extent, the countries of origin of Ethnic German migrants (Gerber and Berman, 2010; Vereshchagina et al., 2015)—display partnership patterns that are in line with the traditional family values of their countries of origin, which is basically characterized by high acceptance of marriage and low acceptance of alternative partnership arrangements. These traditional patterns are transmitted to the next generation.

In contrast to the socialization hypothesis, the *adaptation hypothesis* explains why there might be a convergence in the partnership behaviors of immigrants and the population in the country of destination (Gordon, 1964; Alba and Nee, 1997). The argument stresses the significance of social interactions with the majority population in the country of destination. Thus, moving to a country with less traditional family values may also lead to the adoption of less traditional family-related norms within the migrant population. Given that first-generation immigrants from traditional countries often immigrated as a married couple or in order to get married, their partnership behaviors can hardly be adapted. However, this perspective can help to explain the second generation's partnership behavior (although the idea of a simple assimilation process has been challenged; cf. Portes and Zhou, 1993).

In the literature, *compositional effects* are also thought to explain differences in the behavior of migrants and natives (Bean and Tienda, 1987). Previous studies on marriage patterns have, for example, shown that individuals from lower socio-economic groups marry earlier than individuals from higher socio-economic groups (Oppenheimer, 1997). Although second-generation immigrants attend school longer than first-generation immigrants (Dustmann et al., 2012), the educational differences among native Germans and the second immigrant generation persist (Fick, 2011). Following the composition hypothesis, these educational differences account for differences in the partnership patterns of migrants and natives (Crul and Vermeulen, 2006; Heath et al., 2008).

In addition to socialization, adaptation, and composition, there might be other mechanisms at work that explain the partnership behavior of migrants. Kalmijn (1998) referred to the relevance of opportunity structures for union formation. These structures may be linked to migration in the sense that bringing a partner from abroad to Germany often requires migrants to marry. In Germany, a residence permit is hard to obtain through any means other than marriage, at least for non-EU migrants (Schroedter, 2011, p. 10).

Empirical research analyzing migrants' partnership living arrangements is scarce. Overall, the results of existing studies indicate that the partnership living arrangements of first-generation immigrants from traditional countries, which are characterized by traditional gender norms and a high level of religiosity, who migrated to less traditional countries have a lower incidence of cohabitation, and are more likely than natives to be married [Rahnu et al. (2015) for Russian immigrants in Estonia; Milewski and Hamel (2010) for Turkish immigrants in France; Naderi (2008) for Turkish immigrants in Germany; De Valk and Liefbroer (2007) for immigrants of Turkish and Moroccan origin in the Netherlands; Berrington (1994) for immigrants of South Asian origin in the UK]. These findings offer support for the socialization hypothesis. In addition, second-generation immigrants from traditional countries are more conservative than the native population: compared to natives, they are more likely to be married [Hamel et al. (2012) for Turkish migrants in Germany] have more restrictive attitudes toward cohabitation [Bernhardt et al. (2007) for Turkish migrants in Sweden], and are less likely to expect to cohabit in the future [Berrington (2018) for Black Africans, Indians, Pakistanis, and Bangladeshis in the UK].

## Data

Our analysis draws on German Microcensus data (Destatis, 2019c) from the years 2009 and 2013^2^ (two cross-sections). The German Microcensus is a rotating panel in which respondents are interviewed once per year for four years in a row; i.e., we can pool the two survey years without any repeated observations. The data contain representative information on the social and economic situations of a 1% sample of all households in Germany. The Scientific Use Files that we use in our study contain a 70% subsample of the Microcensus. One of the main advantages of the data is that their large sample size allows us to analyse first-generation Ethnic German immigrants (self-appraisal as Ethnic German immigrant, born in a country other than Germany) and first-generation Turkish (born in Turkey) as well as second-generation Turkish immigrants (with both parents born in Turkey)^3^ as separate groups. We categorize all individuals, who were born in Germany and whose parents are not immigrants, as native Germans. Another advantage of the Microcensus is that nonresponse is of minor relevance because participation is obligatory, and respondents are required by law to submit information. Our sample consists of 6,031 migrant women and 6,007 migrant men (compared to 73,417 native women and 74,814 native men). We focus on respondents between the ages of 18 and 40 because partnerships are most diverse in this age group. Because women tend to marry men who are, on average, two to three years older (Buss, 1989), we analyse men and women separately. Unfortunately, the Microcensus does not include partnership histories. Therefore, we are unable to analyse the transition into a specific partnership living arrangement. Our analyses instead refer to the partnership status of respondents at the time of interview.

While our analysis compares first- and second-generation Turkish immigrants living in Germany, it should be noted that we do not compare migrant parents to their own children. As the German Microcensus is a household survey, we do not have information linking parents and children unless they live in the same household. Because we also want to investigate respondents who live in a household without a partner, we do not take a couple perspective, but rather analyse male and female individuals separately. We excluded respondents residing in the eastern part of the country (except Berlin) because eastern and western Germans still differ in their partnership behaviors (Klärner and Knabe, 2017), and because most immigrants of Turkish origin migrated to western Germany and Berlin and continue to live there (Destatis, 2019d).

We study respondents' partnership living arrangement as our outcome variable, which we categorize as follows: (1) living without a partner in an independent household (including individuals who live in a shared flat); (2) living without a partner in the parental household; (3) living with a partner in a shared household without being married (cohabiting); and (4) living as a married couple in a shared household (married). Categories 1 and 2 include singles, but also individuals in living apart together relationships, as the partnership status in the Microcensus does not refer to partners outside of the household. Categories 3 and 4 includes individuals that live with their partner in their parents' home.

## Methods

In a first step, we report the percentage of partnership arrangements in each immigrant group. In a second step, we estimate multinomial logistic regressions. This allows us to account for potential composition effects. The independent variables included in the multiple regression analysis are age (as a continuous covariate), education (enrolled in education, no degree, vocational degree, university degree), and a year dummy (2009 and 2013). Table 1 reveals that a large share of first-generation Turkish immigrants have no degree. Moreover, the first-generation immigrants are, on average, older than both the natives and the second-generation Turkish migrants in our sample. While about half of the native respondents participated in 2009 and half in 2013, Ethnic Germans and first-generation Turkish immigrants are overrepresented in the 2009 data, and second-generation Turkish immigrants are overrepresented in the 2013 data. This discrepancy is related to the age structure in the immigrant samples. Because most first-generation immigrants arrived in Germany some decades ago, the number of such immigrants who are in the 18–40 age group is getting smaller over time. By contrast, most second-generation Turkish immigrants are still young, with more entering the 18–40 age group over time.

**Table 1:** Descriptive statistics.

	**Native Germans**	**1^**st**^ gen. Ethnic Germans**	**1^**st**^ gen. Turkish**	**2^**nd**^ gen. Turkish**
**FEMALES**				
**Partnership status**				
No partner, independent household	28.6	11.8	2.4	9.6
No partner, parental household	22.2	3.0	1.3	45.3
Cohabiting	15.5	4.1	0.4	1.7
Married	33.8	81.2	95.9	43.5
**Education**				
Enrolled in education	27.8	8.1	2.6	34.3
No degree	8.8	30.9	86.3	23.9
Vocational degree	50.3	52.4	9.3	37.0
University degree	13.2	8.6	1.9	4.8
**Age (mean; standard deviation)**	28.9 (6.7)	33.7 (4.9)	32.7 (5.3)	26.5 (6.2)
**Survey year**				
2009	52.4	58.1	55.6	40.0
2013	47.7	41.9	44.4	60.0
Observations	73,417	2,035	1,515	2,481
**MALES**				
**Partnership status**				
No partner, independent household	30.3	12.2	5.4	11.2
No partner, parental household	31.3	7.5	1.3	54.8
Cohabiting	13.7	4.1	1.5	2.7
Married	24.8	76.2	91.8	31.4
**Education**				
Enrolled in education	29.3	6.1	3.2	34.5
No degree	9.1	30.6	64.1	27.1
Vocational degree	49.4	55.6	26.6	35.6
University degree	12.2	7.7	6.1	2.9
**Age (mean; standard deviation)**	28.9 (6.7)	33.9 (4.7)	33.6 (4.8)	26.6 (6.4)
**Survey year**				
2009	51.9	57.9	57.8	39.6
2013	48.1	42.1	42.2	60.4
Observations	74,814	1,916	1,188	2,903

Column percent and means.

Percentages may not sum to 100 due to rounding.

Source: German Microcensus 2009 and 2013, respondents living in western Germany and Berlin. Respondents between 18 and 40 years old. “No partner” refers to individuals who do not share a household with a partner.

## Results

Our first research question refers to the prevalence of partnership arrangements by immigrant status. Table 1 shows that marriage is the most prevalent living arrangement for Ethnic Germans (81.2% of females and 76.2% of males) and first-generation Turkish immigrants in the 18–40 age group (95.9% of females and 91.8% of males). The other forms of partnership arrangements are marginal in these two immigrant groups. Among Ethnic Germans, the second-largest group is made up of individuals who are living in an independent household without a partner (11.8% of women and 12.2% of men). Among natives, the four partnership types are distributed more equally, with marriage being the most frequent arrangement among women (33.8%), and living in the parental household being the most frequent arrangement among men (31.3%). The most striking difference between the second-generation Turkish respondents and the other three groups is in the likelihood of living in the parental home. Cohabitation seems to be largely unacceptable in the three immigrant groups. It is also the least common living arrangement among natives: 15.5% of native women and 13.7% of native men in our sample are cohabiting. The differences in the prevalence of marriage and of living in the parental home might be associated with the age structure in the four groups, as the mean age of natives and second-generation Turkish in our sample is considerably lower than it is among Ethnic Germans and first-generation Turkish respondents. In Table 3 in Supplementary Material, we describe the partnership arrangements among those in the 18-30 and 31-40 age groups separately. The numbers imply that the differences across groups are related to a higher mean age (as well as a higher mean age at marriage), especially among natives and second-generation Turkish women. In the older age group, marriage is the most prevalent living arrangement among women in all four groups. This is also the case among men, although the share of married men is considerably lower among natives (49.2%) than it is among men in the other three groups. The share of individuals who live in an individual household without a partner is considerably larger among second-generation Turkish immigrants than it is among the first generation, especially in the older age group.

The second aim of our analysis is to assess whether the compositional differences account for variations in partnership arrangements across immigrant groups. In addition to age, education might be associated with partnership choices. We estimate multiple multinomial regressions for men and women. Because the interpretation of the parameters of a multinomial logit model is not straightforward, we present the average marginal effects (AME). The average marginal effect is the mean of the marginal effects for each combination of covariates in the dataset. It represents the average change in the probability of seeing a specific outcome when we alter the respective independent variable from the reference to a different category, based on our sample. The results displayed in Table 2 confirm the patterns reported in our descriptive analyses: i.e., compared to natives, immigrants are more likely to be married, and less likely to be living in an independent household or to cohabit. These findings persist after controlling for education, age, and survey year. A comparison of the AME of immigrant status with and without education as a control (see Table 4 in Supplementary Material) shows that including education in our models only slightly reduces the effect sizes of immigrant status. This finding indicates that the variation in partnership arrangements between immigrant groups and native Germans can be attributed to educational differences to a very limited extent only. In order to determine whether the differences between first- and second-generation Turkish immigrants are significant, we specified additional models with the first generation as the reference category (results not shown here). We found for both males and females that first-generation immigrants were significantly more likely to be married and less likely to be living without a partner in the parental household than second-generation Turkish immigrants.

**Table 2:** Multinomial logistic regression models.

	**Females**				**Males**			
	**No partner, indep. household**	**No partner, parental household**	**Cohabiting**	**Married**	**No partner, indep. household**	**No partner, parental household**	**Cohabiting**	**Married**
**Immigrant status**								
Native Germans	0	0	0	0	0	0	0	0
1^st^ gen. Ethnic German	−0.097***	−0.069***	−0.089***	0.255***	−0.148***	−0.049***	−0.088***	0.285***
1^st^ gen. Turkish	−0.220***	−0.121***	−0.144***	0.485***	−0.189***	−0.231***	−0.109***	0.529***
2^nd^ gen. Turkish	−0.192***	0.125***	−0.137***	0.204***	−0.196***	0.111***	−0.108***	0.193***
**Education**								
Enrolled in education	0.195***	0.089***	−0.053***	−0.231***	0.152***	0.065***	−0.054***	−0.162***
No degree	0.035***	0.006	−0.031***	−0.010	0.077***	0.042***	−0.027***	−0.092***
Vocational degree	0	0	0	0	0	0	0	0
University degree	0.049***	−0.000	0.023***	−0.071***	0.036***	−0.062***	0.030***	−0.004
**Age**	0.004***	−0.029***	−0.003***	0.028***	0.006***	−0.031***	−0.000	0.026***
**Survey year**								
2009	0	0	0	0	0	0	0	0
2013	0.007*	−0.003	0.004	−0.008**	0.015***	−0.003	0.002	−0.014***
BIC		160,247.0				167,163.1		
McFadden's Pseudo *R^2^*		0.25				0.23		
Observations		79,448				80,821		

Average marginal effects.

* p < 0.05;

** p < 0.01;

*** p < 0.001. Predicted probabilities of the reference individual (i.e., native German, vocational degree, 29 years old, survey year 2009): $\hat{p}$(no partner, indep. household; females) = 0.32, $\hat{p}$(no partner, parental household; females) = 0.08, $\hat{p}$(cohabiting; females) = 0.22, $\hat{p}$(married; females) = 0.38; $\hat{p}$(no partner, indep. household; males) = 0.35, $\hat{p}$(no partner, parental household; males) = 0.21, $\hat{p}$(cohabiting; males) = 0.21, $\hat{p}$(married; males) = 0.23.

Source: German Microcensus 2009 and 2013, respondents living in western Germany and Berlin, 18-40 age group. “No partner” refers to individuals who do not share a household with a partner.

For the control variables, the results are largely in line with the literature. Individuals who are enrolled in education are less likely to be married or cohabiting than individuals with a vocational degree. This is also the case for women with a university degree, whereas men with university education do not differ in their likelihood of being married from those with a vocational degree. Highly educated individuals are more likely to cohabit. The AME of having no degree shows an insignificant association with marriage for women, but a significantly negative association for men. For age, the strongest associations are found for marriage and living in the parental home: The older a person is, the more likely s/he is to be married, and the less likely s/he is to be living without a partner in the parental household. There seems to be a slight (but statistically significant) shift in partnership patterns over time, with the probability of living in a marital union being lower and the probability of living in an independent household without a partner being higher in 2013 than in 2009.

## Discussion

Partnership living arrangements are an integral part of the family formation process, and thus greatly affect the lives of adult migrants and natives alike. The findings of this research suggest that these arrangements differ substantially between migrant and native adults in Germany. Marriage is by far the most common partnership form among the Turkish first generation, as well as among Ethnic German immigrants in the 18–40 age group. Among second-generation Turkish immigrants, the most prevalent partnership arrangements are “no partner, living in the parental household” and “married”. Cohabitation seems to be unacceptable in all three immigrant groups, whereas it is a common, albeit infrequent, arrangement among native Germans (15.5% of women, 13.7% of men). Our multiple regression results indicate that these patterns can be explained by differences in educational attainment between migrants and natives to a very small extent only.

Clearly, the higher prevalence of marriage among immigrants is associated with their lower mean age at marriage. The mean age at marriage in Germany is 32.1 years for women and 34.6 years for men (Destatis, 2020). The mean age at marriage among Turkish immigrants in Germany is 24 years, which is the earliest average age among the labor migrant groups in Germany (Schroedter, 2013, p. 205). Apart from a timing effect, it seems plausible to assume that the low prevalence of cohabitation among immigrants is related to traditional family values in the country of origin—which would be in line with the socialization hypothesis. Because of data limitations, we were unable to explicitly account for the role of such traditional values. However, existing research shows, for instance, that compared to respondents from 35 other countries, Turkish respondents express the highest support for marriage (Voicu, 2017). When the same study looked at attitudes in one of the sending countries of Ethnic German immigrants (Poland), respondents expressed more support for conservative family values than Germans. Similarly, a study on family values among adolescents showed that Russians are more traditional than Germans (Mayer et al., 2009). Our finding that almost half of the second-generation Turkish immigrants in our sample are still living with their parents is in line with prior research showing that 68% of Turkish respondents agreed with the statement that children should live with their parents until they get married (Von Gostomski, 2010, p. 208). Although the share of cohabiting individuals remains very low among Turkish second-generation immigrants, we found that they are considerably more likely than first-generation immigrants to be living in an independent household without a partner, especially if they are under age 30. This could be a first sign of the liberalization and adaptation of partnership arrangements in an ethnic group who strictly disapprove of cohabitation.

Another reason for the high prevalence of marriage among Turkish and Ethnic German immigrants is more practical. The immigration of individuals from outside the EU is legally restricted, but the availability of family reunification visas facilitates the migration of the spouses of EU residents, and allows married couples to live together in Germany (Schroedter, 2011). This applies in particular to the residence permits of migrants from Turkey and Russia, one of the most common origin countries of Ethnic German migrants in the last decade (Bundesamt für Migration und Flüchtlinge, 2016a, p. 25). Based on official 2014 visa statistics of the Central Register of Foreigners, Turkey is the most common country of origin for migrant spouses, followed by Russia (Bundesamt für Migration und Flüchtlinge, 2016a, p. 25). This observation is of particular relevance for first-generation immigrants, but also for the relatively high share (32%) of second-generation immigrants of Turkish origin in Germany who have a partner from Turkey (Schroedter, 2011, p. 10).

The major weakness of the present research is related to the limits of the analyzed data, the German Microcensus. First, the information collected in the survey are very basic. In order to explain differences in partnership living arrangements between immigrant groups and generations, information about the partnership context at time of migration, religion, attitudes, and reasons for immigration is needed. The living arrangements of adults may also depend on factors such as the labor, housing market, and economic conditions; the decisions of peers; as well as norms and cultural expectations (Aassve et al., 2013)—none of which are surveyed in the German Microcensus. A second drawback is related to the cross-sectional nature of the data. In order to analyse the dynamic character of partnership formation, future research should use longitudinal data. Given the lack of an appropriate dataset to explain differences in partnership living arrangements by migration generation and origin in the German context, we encourage data collectors to oversample migrant groups and include more partnership- and migration-related items in the question programmes of future longitudinal data projects.

## Data Availability Statement

The datasets generated for this study will not be made publicly available because the German Microcensus is only accessible for registered users.

## Author Contributions

All authors listed have made a substantial, direct and intellectual contribution to the work, and approved it for publication.

## Conflict of Interest

The authors declare that the research was conducted in the absence of any commercial or financial relationships that could be construed as a potential conflict of interest.

## Footnotes

^1^A data source that is frequently used to analyse immigrants' outcomes in Germany is the Socioeconomic Panel (SOEP). However, the number of partnership transitions of separate origin groups in this dataset is too small to allow for meaningful longitudinal analyses. This point is illustrated in a recent study of Turkish immigrants that is based on the SOEP data (Bettin et al., 2018). Among the first generation, the authors observed three partnership transitions (either marriage or transition to cohabitation), and among the second generation, the authors observed nine transitions [calculated from Bettin et al. (2018, p. 1,024)].

^2^In the Scientific Use File of the German Microcensus in the years 2009 and 2013, a number of items allowed us to correctly specify Ethnic Germans and second-generation migrants. In the other years, migrants can be identified only on the basis of citizenship and place of birth; i.e., we were unable to identify naturalized migrants and descendants of migrants who were born in Germany and who had German citizenship.

^3^Children of interethnic parents—i.e., those with one Turkish-born and one native German parent—differ in their integration outcomes from individuals with monoethnic parents (Platt, 2012; Kalmijn, 2015). Because this group was too small to allow for meaningful analyses (0.1% of the total sample), we excluded them from our sample.
