# Supplementary material for: Partnership Living Arrangements of Immigrants and Natives in Germany
Source: Front Sociol. 2020 Nov 13;5:538977. doi: 10.3389/fsoc.2020.538977 (PMC8022747; doi:10.3389/fsoc.2020.538977)
Supplement: Supplementary file 1 [file Data_Sheet_1.docx]

**Appendix**

*Table 3 Partnership status by gender and age group.*

|  | **Native Germans** | | **1^st^ gen.**  **Ethnic German** | **1^st^ gen.**  **Turkish** | **2^nd^ gen.**  **Turkish** |
| --- | --- | --- | --- | --- | --- |
| **Females** |  |  | | | |
| **Partnership status (total)** |  | |  |  |  |
| No partner, independent household | 28.6 | | 11.8 | 2.4 | 9.6 |
| No partner, parental household | 22.2 | | 3.0 | 1.3 | 45.3 |
| Cohabiting | 15.5 | | 4.1 | 0.4 | 1.7 |
| Married | 33.8 | | 81.2 | 95.9 | 43.5 |
| Observations | 73,417 | | 2,035 | 1,515 | 2,481 |
| **Partnership status (age group 18-30)** |  | |  |  |  |
| No partner, independent household | 32.6 | | 23.7 | 3.2 | 8.9 |
| No partner, parental household | 36.2 | | 8.3 | 3.6 | 60.9 |
| Cohabiting | 17.7 | | 9.5 | 0.2 | 1.4 |
| Married | 13.6 | | 58.4 | 92.9 | 28.8 |
| Observations | 42,531 | | 493 | 496 | 1,745 |
| **Partnership status (age group 31-40)** |  | |  |  |  |
| No partner, independent household | 23.2 | | 8.0 | 2.0 | 11.0 |
| No partner, parental household | 2.8 | | 1.2 | 0.2 | 8.6 |
| Cohabiting | 12.5 | | 2.3 | 0.5 | 2.2 |
| Married | 61.6 | | 88.5 | 97.4 | 78.3 |
| Observations | 30,886 | | 1,542 | 1,019 | 736 |
| **Males** |  | | |  | |
| **Partnership status (total)** |  | |  |  |  |
| No partner, independent household | 30.3 | | 12.2 | 5.4 | 11.2 |
| No partner, parental household | 31.3 | | 7.5 | 1.3 | 54.8 |
| Cohabiting | 13.7 | | 4.1 | 1.5 | 2.7 |
| Married | 24.8 | | 76.2 | 91.8 | 31.4 |
| Observations | 74,814 | | 1,916 | 1,188 | 2,903 |
| **Partnership status (age group 18-30)** |  | |  |  |  |
| No partner, independent household | 31.2 | | 25.6 | 9.5 | 9.6 |
| No partner, parental household | 48.7 | | 20.4 | 4.4 | 72.9 |
| Cohabiting | 13.1 | | 8.1 | 2.7 | 1.9 |
| Married | 7.1 | | 46.0 | 83.5 | 15.6 |
| Observations | 43,380 | | 446 | 296 | 2,024 |
| **Partnership status (age group 31-40)** |  | |  |  |  |
| No partner, independent household | 29.0 | | 8.2 | 4.0 | 14.8 |
| No partner, parental household | 7.3 | | 3.6 | 0.2 | 13.1 |
| Cohabiting | 14.5 | | 2.9 | 1.1 | 4.6 |
| Married | 49.2 | | 85.4 | 94.6 | 67.6 |
| Observations | 31,434 | | 1,470 | 892 | 879 |

Column percent.

Note: Percentages may not sum to 100 due to rounding.

*Source: German Microcensus 2009 and 2013, respondents living in western Germany and Berlin, 18-40 age group. “No partner” refers to individuals who do not share a household with a partner.*
