# Supplementary material for: Partnership Living Arrangements of Immigrants and Natives in Germany
Source: Front Sociol. 2020 Nov 13;5:538977. doi: 10.3389/fsoc.2020.538977 (PMC8022747; doi:10.3389/fsoc.2020.538977)
Supplement: Supplementary file 2 [file Data_Sheet_2.docx]

*Table 4 Multinomial logistic regression model. Average marginal effects. Without education.*

|  | **Females** | | | | **Males** | | | |
| --- | --- | --- | --- | --- | --- | --- | --- | --- |
|  | **No partner, indep. household** | **No partner, parental household** | **Cohabiting** | **Married** | **No partner, indep. household** | **No partner, parental household** | **Cohabiting** | **Married** |
| **Immigrant status** |  |  |  |  |  |  |  |  |
| Native Germans | 0 | 0 | 0 | 0 | 0 | 0 | 0 | 0 |
| 1^st^ gen. Ethnic German | -0.102^***^ | -0.074^***^ | -0.094^***^ | 0.270^***^ | -0.143^***^ | -0.044^***^ | -0.089^***^ | 0.276^***^ |
| 1^st^ gen. Turkish | -0.240^***^ | -0.159^***^ | -0.147^***^ | 0.546^***^ | -0.189^***^ | -0.234^***^ | -0.109^***^ | 0.532^***^ |
| 2^nd^ gen. Turkish | -0.193^***^ | 0.120^***^ | -0.138^***^ | 0.212^***^ | -0.189^***^ | 0.123^***^ | -0.109^***^ | 0.175^***^ |
| **Age** | 0.000 | -0.034^***^ | 0.001^***^ | 0.033^***^ | 0.003^***^ | -0.035^***^ | 0.003^***^ | 0.030^***^ |
| **Survey year** |  |  |  |  |  |  |  |  |
| 2009 | 0 | 0 | 0 | 0 | 0 | 0 | 0 | 0 |
| 2013 | 0.013^***^ | 0.000 | 0.004 | -0.017^***^ | 0.016^***^ | -0.005 | 0.003 | -0.015^***^ |
| BIC | 163659.5 | | | | 169552.1 | | | |
| McFadden’s  Pseudo R^2^ | 0.23 | | | | 0.22 | | | |
| Observations | 79,448 | | | | 80,821 | | | |

Note: ^*^ p<0.05; ^**^p<0.01; ^***^p<0.001. Predicted probabilities of the reference individual (i.e., native German, 29 years old, survey year 2009): p̂(no partner, indep. household; females)=0.38, p̂(no partner, parental household; females)=0.09, p̂(cohabiting; females)=0.21, p̂(married; females)=0.32; p̂(no partner, indep. household; males)=0.41, p̂(no partner, parental household; males)=0.22, p̂(cohabiting; males)=0.18, p̂(married; males)=0.19.

*Source: German Microcensus 2009 and 2013, respondents living in western Germany and Berlin, 18-40 age group. “No partner” refers to individuals who do not share a household with a partner.*
